# Disopyramide for Hypertrophic Cardiomyopathy

**DOI:** 10.7759/cureus.4526

**Published:** 2019-04-23

**Authors:** Alejandro Sanchez-Nadales, Andrea Anampa-Guzmán, Amir Khan

**Affiliations:** 1 Internal Medicine, Advocate Illinois Masonic Medical Center, Chicago, USA; 2 Internal Medicine, Main National University of San Marcos, Lima, PER

**Keywords:** myosin, cardiomyopathy, hypertrophic

## Abstract

Hypertrophic cardiomyopathy (HCM) is a cardiac disease characterized by hypertrophy of a nondilated left ventricle without any other cardiac or systemic disease that could account for observed hypertrophy. We present a female patient with the diagnosis of obstructive HCM, which is rare because sudden death is frequently the first clinical presentation. Her symptoms were resistant to medical treatment and the patient did not want invasive procedures. Disopyramide was added, and it improved her symptoms. Disopyramide is a safe and effective medication that reduces symptoms and delays the need for invasive therapy. Disopyramide was added to the treatment of the patient and it improved her symptoms.

## Introduction

Hypertrophic cardiomyopathy (HCM) is a cardiac disease characterized by hypertrophy of a nondilated left ventricle without any other cardiac or systemic disease that could account for observed hypertrophy [1]. HCM is caused by genetic mutations in both the thick and thin contractile myofilament proteins of the cardiac sarcomere. There are currently eight myofilament-encoding genes that are recognized as hypertrophic cardiomyopathy-associated susceptibility genes: cardiac myosin binding protein C (MYBPC3), beta-myosin heavy chain (MYH7), myosin essential light chain (MYL2), myosin regulatory light chain (MYL3), cardiac troponin T (TNNT2), cardiac troponin I (TNNI3), alpha-tropomyosin (TPM1) and cardiac alpha-actin (ACTC1) [2].

HCM affects about 0.2% in the general population. The estimated annual incidence of hypertrophic cardiomyopathy is 0.47 per 100,000 children and adolescents in New England and Central Southwest regions of the United States [3]. There are two types of HCM; Obstructive HCM, characterized by the presence of left ventricular outflow tract obstruction, constitutes about 70% of cases. The remaining 30% of cases are the nonobstructive form, which lacks this characteristic. Half of the patients with the obstructive form have septal hypertrophy (obstruction at rest with ≥30 mm Hg gradient), and the other half have a labile obstruction (obstruction with provocation and <30 mm Hg gradient at rest, but ≥30 mm Hg gradient with physiologic provocation) [4].

Though patients with HCM are often asymptomatic, symptoms include chest pain associated with exertional dyspnea, palpitations due to atrial or ventricular arrhythmia, lightheadedness and syncope. This is often accompanied by a systolic murmur [4,5]. The complications of HCM are syncope, heart failure, and sudden death [1].

## Case presentation

**Figure 1:**
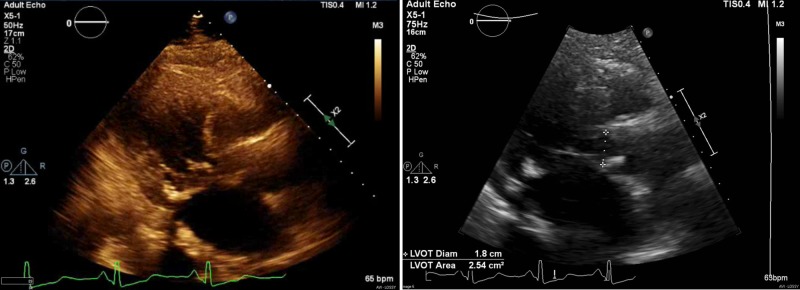
Echocardiogram images

A 78-year-old Romanian female with a past medical history of obstructive HCM, moderate aortic stenosis, non-obstructive coronary artery disease, diabetes mellitus type II diet controlled and macular degeneration came to the cardiology consult. The patient has moderate to severe wall hypertrophy and significant left ventricular outflow tract (LVOT) gradient that is difficult to grade but appears greater than 50 mm Hg (Figure 1). Systolic function is normal. She complains of occasional palpitations, cough, and dyspnea on mild activities. The patient had moderate aortic stenosis. The patient accepted medicine but declined any intervention. Edema is present in the lower extremities, and the patient claims that it has been present for over four weeks. The patient was transferred to the emergency department due to worsening shortness of breath. The patient said she cannot walk for more than half a block. The patient was on metoprolol ER 100 mg because she could not tolerate a verapamil trial in the past. Due to this, it was decided to start disopyramide given her worsening symptoms.

## Discussion

We presented a female patient with the diagnosis of obstructive HCM, which is rare because sudden death is frequently the first clinical presentation [4]. The initial testing in patients suspected of having HCM is a 12-lead electrocardiogram (ECG) and transthoracic echocardiography (TTE). Left ventricular hypertrophy in adults with HCM is commonly defined as a maximal left ventricular free wall thickness ≥15 mm, with 13-14 mm considered borderline. Patients with HCM are advised to refrain from participating in intense competitive sports. However, they can do low intensity noncompetitive aerobic exercise as a component of a healthy lifestyle if asymptomatic [6].

Symptomatic patients are treated with beta blockers to treat angina or dyspnea in adults. Physicians should titrate this dose to achieve a resting heart rate of 60-65 beats per minute in patients who do not respond to low doses of beta blockers for angina or dyspnea. Verapamil should be used to treat angina or dyspnea in patients who do not respond to beta blockers, experience side effects, or have contraindications, and caution should be used when treating patients with high outflow gradients, heart failure, or sinus bradycardia [6]. The patient in our case did not respond to beta blockers or verapamil. Septal ablation therapies are done in patients with medication-refractory symptoms. However, our patient did not want to have procedures and was put on disopyramide. It is recommended to consider adding disopyramide to a beta blocker or verapamil therapy to treat angina or dyspnea if there is no response to these treatments alone. This should be avoided in patients with reduced systolic function [7].

Disopyramide is a class IA antiarrhythmic drug. It is a sodium channel blocker with negative inotropic properties that effectively reduces left ventricular outflow tract gradients in adults with HCM [8]. Patients with HCM who are persistently symptomatic despite the use of β-blockers or verapamil and have evidence of LVOT obstruction are prescribed disopyramide [7]. Disopyramide could improve LV diastolic dysfunction in patients with hypertrophic nonobstructive cardiomyopathy (HNCM) [9]. Sherrid et al. reported the long-term efficacy and safety of disopyramide in patients with obstructive HCM in a multicenter study. Two-thirds of the patients treated with disopyramide could be managed medically with amelioration of symptoms and about 50% reduction in the subaortic gradient for over three years [10]. Disopyramide is a safe and effective medication that reduces symptoms, LVOT gradient and delays the need for invasive therapy [11]. Patients with HCM have an average life expectancy with little or no disability without need for major therapeutic interventions. Most patients with nonobstructive hypertrophic cardiomyopathy do not advance to progressive heart failure [1].

## Conclusions

HCM is a disease where the myocardium has an abnormal thickening. The thickening of the heart muscle can make it difficult for the latter to pump blood. HCM is often asymptomatic. However, it can cause shortness of breath, chest pain and arrhythmias. We presented the case of a woman with symptoms resistant to medical treatment who did not want invasive procedures. Disopyramide was added, and it improved her symptoms.
